# Status Epilepticus in a Patient Prescribed Theophylline: A Case Report

**DOI:** 10.5811/cpcem.61649

**Published:** 2026-10-06

**Authors:** Cynthia Coyle, Emmelyn J. Samones, Tammy Phan, Mindi Guptill

**Affiliations:** Loma Linda University Medical Center, Department of Emergency Medicine, Loma Linda, California

**Keywords:** seizure, theophylline, toxicity, status epilepticus, case report

## Abstract

**Introduction:**

Seizures are a frequent reason for emergency department (ED) evaluation, and new-onset events carry a wide differential in which medication toxicity can be overlooked. Toxicity from seldomly prescribed drugs, such as theophylline, can be particularly challenging to recognize because they can cause nonspecific adverse effects, including palpitations, nausea, vomiting, and seizures. Although theophylline use was widespread in the 1980s, current prescribing patterns are unknown. Nevertheless, theophylline toxicity continues to result in ED visits and poison center calls. Because adverse effects may be acute or chronic, identifying theophylline toxicity remains difficult.

**Case Report:**

A 74-year-old woman presented to the ED with status epilepticus. After thorough history and extensive medical work-up, medication toxicity was suspected as the cause of her seizures. Upon further investigation, the patient was found to have theophylline toxicity. Prompt treatment was initiated resulting in cessation of seizures, highlighting the importance of considering medication toxicity in geriatric patients with new-onset seizures.

**Conclusion:**

Theophylline toxicity can present with vague, multisystem symptoms, with status epilepticus being one of its most severe complications. Rapid identification of the underlying toxic etiology is essential to guide treatment and stop the seizures. Moreover, clinical suspicion should be elevated in the geriatric population related to frequent polypharmacy. In this case, confirming the medication history with a family member raised concerns for medication misuse, enabling timely intervention. Although rarely prescribed, physicians should counsel patients on the risks and adverse effects of theophylline and emphasize close monitoring.

## INTRODUCTION

New-onset seizure is a common emergency department (ED) presentation, and the differential is broad and varied.^1^ While head trauma, hypoglycemia, metabolic derangements, and infection are common culprits, medication toxicity can be overlooked.^2^ Toxicity secondary to infrequently prescribed drugs remains a difficult diagnosis. A study done in the ED found that of new-onset seizures presenting to the ED, 6.1% were medication related.^3^ Theophylline was once among the top 20 most prescribed drugs with eleven million annual prescriptions in the 1980s.^3^ However, usage has sharply declined, and no data are currently available on the prescription frequency for theophylline.^4^ While considered an outdated medication and rarely prescribed, theophylline toxicity still accounts for ED visits and poison center calls. In 2022 and 2023, 244 U.S. poison center cases involved theophylline with one death each year attributed to the medication.^5^,^6^ In geriatric patient populations in particular, medication side effects should always be considered, and a thorough medication review should be performed.

## CASE REPORT

A 74-year-old female with past medical history of chronic obstructive pulmonary disease, atrial fibrillation, and heart failure with reduced ejection fraction presented via emergency medical services (EMS) with altered mental status. The patient’s spouse reported decreased responsiveness for approximately five hours prior to presentation. At home, the patient experienced five seizures without return to baseline prompting the spouse to call EMS. Emergency medical services noted the patient having active tonic-clonic seizures during their evaluation and administered two doses of midazolam 2.5 mg intravenously (IV). Upon arrival to the ED, the patient was actively seizing. Vitals obtained upon arrival revealed the patient to be hypertensive to 166/87 mm Hg, tachycardic to 151 beats per minute, and saturating well on supplemental oxygen via nasal cannula. On physical exam, there was no external evidence of trauma or injury.

The patient received two doses of lorazepam 4 mg IV and levetiracetam loading dose (4,200 mg) IV without resolution in seizure activity. The patient was intubated for airway protection and started on a propofol infusion. A point-of-care glucose obtained upon arrival was negative for hypoglycemia. A full medical work-up was notable for a negative urine drug screen with the exception of benzodiazepines (likely related to medication given by EMS and in the ED), and no emergent findings on complete blood count, creatine kinase, comprehensive metabolic panel, troponins, lactate, or urinalysis. A computed tomography of the head was without evidence of acute intracranial abnormality.

On review of her medication list, the patient was noted to be on multiple medications, including digoxin and theophylline, raising concerns for medication toxicity as the cause of her seizures. Therefore, digoxin and theophylline levels were obtained. The digoxin level was undetectable at < 0.4 ng/mL (therapeutic range 0.5–1.0 ng/mL) and the theophylline level was elevated at 21.5 μg/mL (8–16 μg/mL; toxic range > 20 μg/mL). At that time, the working diagnosis was theophylline toxicity as the cause of her seizures.

Further history obtained from the patient’s husband revealed that approximately three weeks prior she had been evaluated in the ED for altered mentation and new-onset seizure activity. She had a witnessed seizure in the ED at that visit. She was given IV levetiracetam in the ED and admitted to the neurology service for new-onset seizures. During that admission, video electroencephalogram did not show evidence of seizure activity. She was subsequently discharged home with a prescription for levetiracetam 1,500 mg per os twice a day.

*CPC-EM Capsule*

What do we already know about this clinical entity?

*The pharmacological agent theophylline has a narrow therapeutic index, and toxicity can cause severe complications, including status epilepticus.*

What makes this presentation of disease reportable?

*This case is an uncommon modern presentation of status epilepticus secondary to theophylline toxicity in a geriatric patient.*

What is the major learning point?

*Medication toxicity, including from infrequently prescribed drugs, should remain on the differential for seizures, especially in the geriatric population.*

How might this improve emergency medicine practice?

*This case highlights the need to obtain a thorough history, including medication reconciliation, to identify toxic etiologies and guide timely, targeted treatment.*

The husband also revealed that the patient was taking greater than her prescribed amount of theophylline 300 mg twice a day for the prior month. It was unclear how much the patient was taking; however, the patient’s husband recalled the patient doubling or tripling her dose due to a cough she had for the prior month. Poison control was consulted and recommended activated charcoal 1 g/kg with serial theophylline levels every four hours (Figure). The patient was admitted to the neurocritical care unit for status epilepticus.

After admission to the neurocritical care unit, electroencephalogram was initiated and IV levetiracetam was continued. Video electroencephalogram showed diffuse background slowing but no seizures or epileptiform discharges. During her 1.5 month hospitalization, the patient developed multiple complications including gastrointestinal (GI) bleeding and atrial fibrillation with rapid ventricular response. During her hospital course, she had issues with secretion management and dysphagia. She required recurrent intubations for airway protection for altered mentation. The patient’s altered mentation was thought to be multifactorial but was not suspected to be secondary to waxing and waning methylxanthine toxicity. The patient was ultimately discharged home on hospice from the intensive care unit.

## DISCUSSION

Theophylline was originally developed in 1922 as a treatment for asthma due to its effect of bronchial dilation and smooth muscle relaxation through β2-adrenergic agonism and increased intracellular cyclic adenosine monophosphate.^7^,^8^ Theophylline works through two separate mechanisms of action; it is both a nonselective adenosine receptor antagonist and a competitive nonselective phosphodiesterase inhibitor, leading to the adverse effects on various systems outside the respiratory system. Theophylline has a narrow therapeutic window and when levels become toxic, most commonly with plasma level > 20 mg/L, adverse effects on the central nervous system, GI system, and the cardiovascular system can occur. Nonspecific symptoms such as GI distress have also been attributed to chronic theophylline overdose, such as the case of a 69-year-old man with chronic obstructive pulmonary disease who presented for medical care with chronic nausea and vomiting. Following an extensive but unrevealing GI evaluation, symptoms were attributed to theophylline toxicity after he was found to have a level of 28.9 μg/mL.^9^

Treatment for theophylline toxicity includes activated charcoal per os or via nasogastric tub. Initial seizure treatment with benzodiazepines is appropriate; however, it may be insufficient due to theophylline’s adenosine antagonism. In the case of refractory seizures, escalation to phenobarbital or propofol infusion may be required.^8^ Hemodialysis is indicated in the unstable patient with life-threatening arrhythmias or seizures. Hemodialysis may also be necessary for theophylline plasma level greater than 100 mg/L or persistent high levels despite appropriate care.

## CONCLUSION

Theophylline toxicity can cause a myriad of nonspecific symptoms involving multiple body systems (neurologic, cardiovascular, respiratory, and GI). Status epilepticus is among the most dangerous adverse effects of theophylline toxicity. Prompt recognition of the underlying cause of the status epilepticus must be identified to address the toxicity and resolve the seizures. Involving the patient’s family member to confirm a medication list raised suspicion for theophylline misuse and toxicity, which allowed for prompt treatment. In the case of new-onset seizures or status epilepticus, toxicity of drugs, such as theophylline, should always be on the physician’s differential, and a thorough review of a patient’s medication list is critical.

## Figures and Tables

**Figure:**
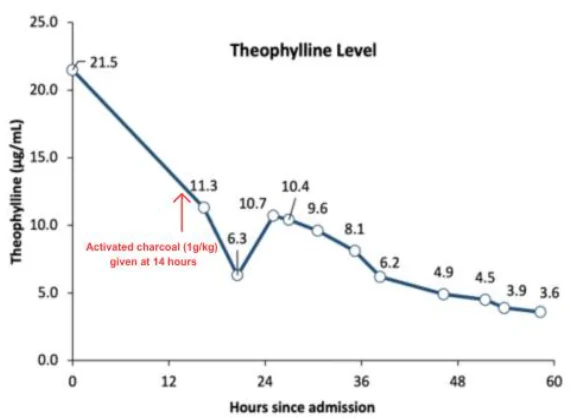
Graph of serial theophylline levels in a 74-year-old female with theophylline toxicity, measured every four hours over a three-day hospital admission. *μg*, micrograms; *mL*, milliliter; *g*, grams; *kg*, kilograms.
